# Supplementary material for: Fever, Tachypnea, and Monocyte Distribution Width Predicts Length of Stay for Patients with COVID-19: A Pioneer Study
Source: J Pers Med. 2022 Mar 12;12(3):449. doi: 10.3390/jpm12030449 (PMC8953796; doi:10.3390/jpm12030449)
Supplement: Supplementary file 1 [file jpm-12-00449-s001.zip › jpm-1637904-supplementary.pdf]

Supplemental Materials

Supplemental Figures: Figures S1-S2

Supplemental Tables: Tables S1-S7

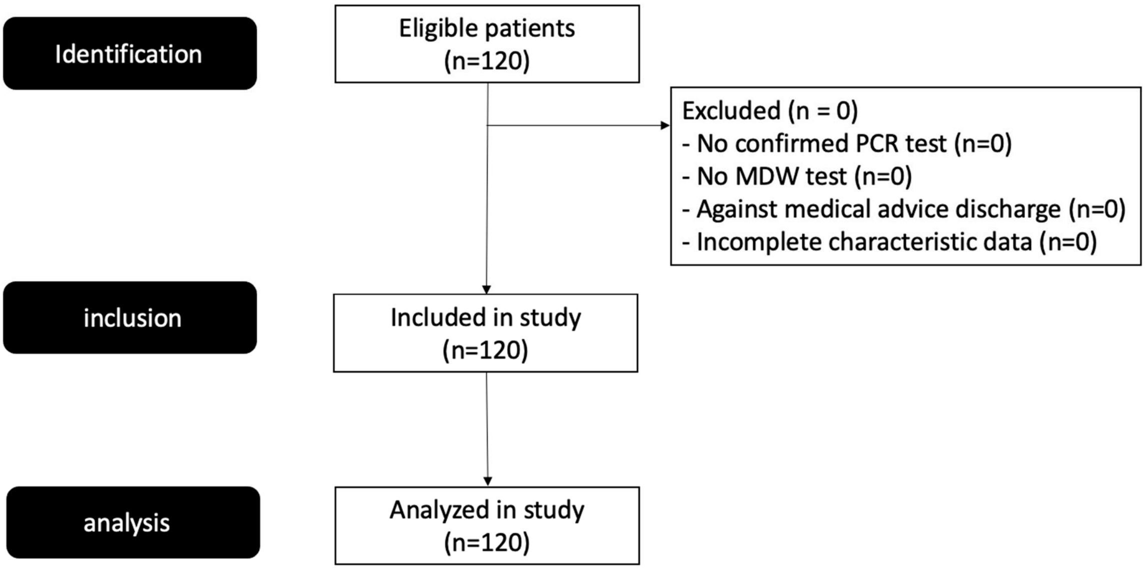

Supplemental Figure S1. The flow diagram.

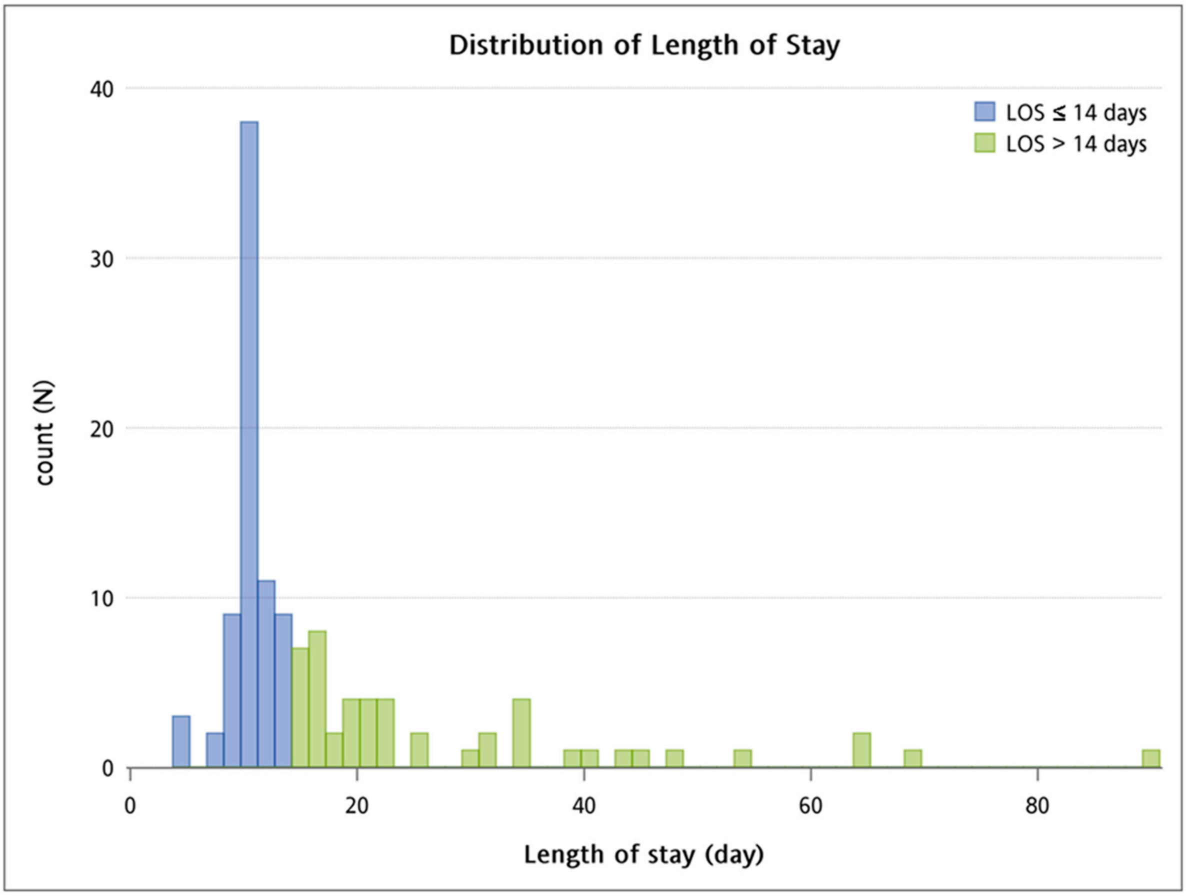

Supplemental Figure S2. The distribution of length of stay (LOS) in our enrolled patients.

Table S1. Clinical spectrum of coronavirus disease 2019 (COVID-19) infection.

Clinical Spectrum	Clinical Presentation	Dyspnea	Chest x-ray	SpO_2	Shock
Mild illness	Patients had any symptoms and signs of COVID-19 (including fever, cough, sore throat, diarrhea, and loss of taste or smell) but did not exhibit shortness of breath, dyspnea	No	Normal	≥94% at room air	No
Moderate illness	Patients had any symptoms and signs of COVID-19 and lower respiratory infection with an oxygen saturation (SpO_2) of ≥94% at room air	Presence	Infiltrates ≤ 50%	≥94% at room air	No
Severe illness	Patients had any above symptoms and signs of COVID-19 with an SpO_2 of <94% at room air, a ratio of arterial partial pressure of oxygen to fraction of inspired oxygen (PaO_2/FiO_2) of <300 mmHg, a respiratory rate of >30 breaths/min, or a chest X-ray with lung infiltrates >50%	Presence, or a respiratory rate of >30 breaths/min	Infiltrates >50%	<94% at room air, or (PaO_2/FiO_2) of <300 mmHg	No
Critical illness	Patients with any above symptoms and signs and exhibited respiratory failure, septic shock, or multiple organ failures	Presence	Infiltrates >50%	<94%, or (PaO_2/FiO_2) of <300 mmHg	Presence

Abbreviations: SpO_2, peripheral capillary oxygen saturation

Table S2. Patient characteristics (*N* = 120).

Characteristic	LOS ≤ 14 days (*N* = 72)	LOS > 14 days (*N* = 48)	*P* value
Age groups (years)			0.0827
17–29	8/72 (11.1%)	2/48 (4.2%)	
30–39	11/72 (15.3%)	2/48 (4.2%)	
40–49	11/72 (15.3%)	6/48 (12.5%)	
50–59	17/72 (23.6%)	8/48 (16.7%)	
60–69	14/72 (19.4%)	14/48 (29.2%)	
70–79	7/72 (9.7%)	11/48 (22.9%)	
≥80	4/72 (5.6%)	5/48 (10.4%)	
Symptoms			
Chest pain	9/72 (12.5%)	3/48 (6.3%)	0.2636
Vital signs at ED			
SBP (mmHg)	131.1 ± 19.2	132.0 ± 22.9	0.8132
DBP (mmHg)	80.1 ± 14.7	78.5 ± 14.7	0.5658
MAP (mmHg)†	97.1 ± 14.6	96.3 ± 15.4	0.9340
Chest X-ray at ED			0.0523
No infiltrate	28/72 (38.9%)	10/48 (20.8%)	
Unilateral infiltrate	9/72 (12.5%)	4/48 (8.3%)	
Bilateral infiltrate	35/72 (48.6%)	34 (70.8%)	
Medical comorbidity			
Hypertension	21/72 (29.2%)	24/48 (50.0%)	0.0209*
Diabetes mellitus	17/72 (23.6%)	11/48 (22.9%)	0.9298
Coronary artery disease	9/72 (12.5%)	12/48 (25.0%)	0.0775
Malignancy	3/72 (4.2%)	4/48 (8.3%)	0.3401
Severity at ED			<0.0001*
Mild	39/72 (54.2%)	9/48 (18.8%)	
Moderate	25/72 (34.7%)	11/48 (22.9%)	
Severe	3/72 (4.2%)	10/48 (20.8%)	
Critical	5/72 (6.9%)	18/48 (37.5%)	

Abbreviations: DBP, diastolic blood pressure; ED, emergency department; ICU, intensive care unit; MAP, mean arterial pressure; SBP, systolic blood pressure;

*Statistical significance ($P < 0.05$).

†The Mann-Whitney U test was used.

Table S3. Univariable predictors of length of stay >14 days (*N* = 120).

**Characteristic**	**PPV (95% CI)**	**NPV (95% CI)**	**Accuracy (95% CI)**
Age > 60 years	55.5% (45.6–65.5%)	72.1% (63.7–79.1%)	65.0% (55.8–73.5%)
Fever > 38°C	62.5% (47.4–75.5%)	68.2% (62.3–73.6%)	66.7% (57.5–75.0%)
Tachypnea > 20 breaths/min	70.8% (52.2–84.4%)	67.7% (62.7–72.4%)	68.3% (59.2–76.5%)
SpO_2 < 96%	52.3% (40.8–63.6%)	67.1% (60.0–73.5%)	61.7% (52.4–70.4%)
MDW ≥ 21	45.9% (42.1–49.7%)	90.9% (71.0–97.6%)	54.2%(44..8–63.3%)
CRP > 3 mg/dL	50.0% (41.0–59.0%)	69.4% (60.3–77.1%)	60.0%(50.7–68.8%)
NLR > 3	48.7% (42.5–55.0%)	76.2% (63.5–85.5%)	58.3%(49.0–67.3%)

Abbreviations: CRP, C-reactive protein; MDW, monocyte distribution width; PPV, positive predictive value; NPV, negative predictive value; SpO_2, peripheral capillary oxygen saturation;

Table S4. Assessment of continuous variable distribution (N = 120).

Variables	Minimum	Maximum	Mean ± SD	Shapiro–Wilk test	
				Statistics	*P* value
Age	17	91	56.5 ± 17.0	0.9775	0.0417*
BMI (kg/m2)	17.6	42.5	25.2 ± 5.0	0.9219	<0.0001*
Body temperature (°C)	35.0	39.2	37.2 ± 0.9	0.9511	<0.0001*
Heart rate (beats/min)	48	134	90.0 ± 17.1	0.9833	0.1451
Respiratory rate (breaths/min)	12	32	19.5 ± 3.6	0.8257	<0.0001*
SpO2 (%)	63	100	95.3 ± 5.1	0.7404	<0.0001*
SBP (mmHg)	78	197	131.5 ± 20.7	0.9795	0.0633
DBP (mmHg)	51	128	79.4 ± 14.7	0.9809	0.0870
MAP (mmHg)	60	151	96.8 ± 14.9	0.9773	0.0401*
SIRS score	0	4	1.5 ± 0.9	0.8793	<0.0001*
qSOFA score	0	3	0.28 ± 0.55	0.5540	<0.0001*
LOS (days)	4	90	17.8 ± 13.8	0.6605	<0.0001*
WBC (10^3 cells/μL)	2.6	19.9	6.5 ± 2.7	0.8787	<0.0001*
CRP (mg/dL)	0.1	31.8	5.2 ± 5.7	0.8299	<0.0001*
PCT (ng/mL)	0.03	7.89	0.33 ± 1.01	0.3058	<0.0001*
MDW	15.9	42.2	24.7 ± 4.5	0.9616	<0.0001*
RDW (%)	12.1	21.1	13.6 ± 1.2	0.7364	<0.0001*
NLR	0.9	42.5	6.0 ± 6.9	0.6593	<0.0001*
PLR	39.7	1144.5	216.0 ± 166.9	0.7031	<0.0001*
Charlson Comorbidity Index	0	8	2.2 ± 2.0	0.8930	<0.0001*
Ct number	12	36	22.7 ± 6.0	0.9536	0.0006*

Abbreviations: CRP, C-reactive protein; Ct, cycle threshold value for COVID-19 positive cases; DBP, diastolic blood pressure; ED, emergency department; ICU, intensive care unit; LOS, length of stay; MAP, mean arterial pressure; MDW, monocyte distribution width; NLR, neutrophil-to-lymphocyte ratio; PCT, procalcitonin; PLR, platelet-to-lymphocyte ratio; qSOFA, the quick Sequential Organ Failure Assessment; SBP, systolic blood pressure; SD, standard deviation; SIRS, systemic inflammatory response syndrome; SpO_2, peripheral capillary oxygen saturation.
*Statistical significance ($P < 0.05$).

Table S5. Multivariable models with and without adjustment of the Charlson Comorbidity Index for predicting length of stay >14 days (N = 120).

Models	With adjustment on CCI		Without adjustment on CCI	
Characteristic	Model 1[a] OR (95% CI)	*P* value	Model 1[a] OR (95% CI)	*P* value
Variable				
Age > 60 years	1.85 (0.55–6.19)	0.3208	1.63 (0.64–4.14)	0.3018
Sex (male vs. female)	1.48 (0.60–3.67)	0.4001	1.45 (0.59–3.57)	0.4188
BT > 38°C	2.46 (0.92–6.55)	0.0717	2.52 (0.96–6.63)	0.0609
RR > 20/min	3.74 (1.12–12.54)	0.0320*	3.60 (1.11–11.64)	0.0329*
SpO_2 < 96%	0.78 (0.28–2.20)	0.6356	0.81 (0.29–2.22)	0.6764
Hypertension	1.55 (0.60–4.01)	0.3720	1.48 (0.59–3.72)	0.4002
MDW ≥ 21	4.72 (0.92–24.15)	0.0624	4.66 (0.92–23.75)	0.0639
CRP > 3 mg/dL	0.88 (0.29–2.69)	0.8274	0.86 (0.29–2.56)	0.7824
NLR < 3	1.68 (0.54–5.22)	0.3705	1.74 (0.57–5.30)	0.3287
CCI	0.95 (0.70–1.30)	0.7545	-	-
Model fit				
AUC (95% CI)	0.787 (0.701–0.874)		0.786 (0.700–0.872)	
AIC	161.68		161.68	
Hosmer–Lemeshow test	8.785 (10 groups)	0.3607	9.106 (10 groups)	0.3334
IDI test	Reference	-	-0.06%	0.5162

Abbreviations: AUC, area under curve; BT, body temperature; CCI, Charlson Comorbidity Index; CI, confidence interval; CRP, C-reactive protein; NLR, neutrophil-to-lymphocyte ratio; OR, odds ratio; RR, respiratory rate; AIC, Akaike information criterion.

[a]Model 1 included all significant predictors from the multivariable analysis.

*Statistical significance ($P < 0.05$).

Table S6. Test of Model 3 in Subgroups of Non-ICU and ICU categories.

	LOS ≤ 14 days	LOS > 14 days	OR (95% CI)	*P* value	AUC (95% CI)
Non-ICU category (*N* = 85)					
Model 3					
Score (per score)	-	-	1.78 (1.12-2.83)	0.0142*	0.693 (0.565-0.812)
Score ≥ 4	10/64 (15.6%)	9/21 (42.9%)	4.05 (1.35-12.12)	0.0124*	0.636 (0.519-0.754)
ICU category (*N* = 35)					
Model 3					
Score (per score)	-	-	1.21(0.59-2.50)	0.6034	0.560 (0.365-0.755)
Score ≥ 4	6/8 (75.0%)	19/27 (70.4%)	0.79 (0.13-4.79)	0.7993	0.523 (0.340-0.706)

Abbreviations: AUC, area under curve; CI, confidence interval; ICU, intensive care unit; LOS, length of stay; OR, odds ratio. *Statistical significance ($P < 0.05$).

Table S7. Test of Newly Developed Model for Independent set of Patients (*N* = 37).

	LOS ≤ 14 days (N = 23)	LOS > 14 days (N= 14)	OR (95% CI)	*P* value	AUC (95% CI)
Model 3					
Score (per score)			1.54 (0.97-2.45)	0.0686	0.655 (0.490-0.820)
Score ≥ 0	17/23 (73.9%)	6/14 (42.9%)			
Score ≥ 1	0/23 (0%)	0/14 (0%)			
Score ≥ 2	0/23 (0%)	1/14 (7.1%)			
Score ≥ 3	6/23 (26.1%)	6/14 (42.9%)			
Score ≥ 4	0/23 (0%)	1/14 (7.1%)			
Score ≥ 5	0/23 (0%)	0/14 (0%)			
Score ≥ 6	0/23 (0%)	0/14 (0%)			

Abbreviations: AUC, area under curve; CI, confidence interval; ICU, intensive care unit; LOS, length of stay; OR, odds ratio.*Statistical significance ($P < 0.05$).
